# Culture of vibrating microtome tissue slices as a 3D model in biomedical research

**DOI:** 10.1186/s13036-023-00357-5

**Published:** 2023-06-01

**Authors:** Fatina Siwczak, Charlotte Hiller, Helga Pfannkuche, Marlon R. Schneider

**Affiliations:** Institute of Veterinary Physiology, University of Leipzig, An den Tierkliniken 7, 04103 Leipzig, Germany

**Keywords:** 3D models, Organotypic culture, Tissue slices, Vibrating microtomes, Microfluidics

## Abstract

The basic idea behind the use of 3-dimensional (3D) tools in biomedical research is the assumption that the structures under study will perform at the best in vitro if cultivated in an environment that is as similar as possible to their natural in vivo embedding. Tissue slicing fulfills this premise optimally: it is an accessible, unexpensive, imaging-friendly, and technically rather simple procedure which largely preserves the extracellular matrix and includes all or at least most supportive cell types in the correct tissue architecture with little cellular damage. Vibrating microtomes (vibratomes) can further improve the quality of the generated slices because of the lateral, saw-like movement of the blade, which significantly reduces tissue pulling or tearing compared to a straight cut. In spite of its obvious advantages, vibrating microtome slices are rather underrepresented in the current discussion on 3D tools, which is dominated by methods as organoids, organ-on-chip and bioprinting. Here, we review the development of vibrating microtome tissue slices, the major technical features underlying its application, as well as its current use and potential advances, such as a combination with novel microfluidic culture chambers. Once fully integrated into the 3D toolbox, tissue slices may significantly contribute to decrease the use of laboratory animals and is likely to have a strong impact on basic and translational research as well as drug screening.

## The dimensions of biomedical research tools

Researchers address their experimental questions by employing a variety of models, which lie on a wide spectrum in terms of tractability and physiological relevance. Animal studies, at one pole, provide valuable insights into the pathophysiology of most biological process in a (manipulable) systemic environment. On the downside, animal experiments can be expensive, time-consuming, technically challenging, and their translational value may be severely compromised by intrinsic species-specific differences, not to mention the substantial ethical dilemma of causing animal pain or suffering. At the other end of the scale, simple two-dimensional cell culture systems are unbeatable in terms of convenience, accessibility, and readout throughput. Regrettably, they fail short in terms of physiological significance due to the lack of essential features typical for the in vivo situation, such as the presence of extracellular matrix (ECM) and proper spatial and signal-based interactions with other cells or tissues. It is therefore not surprising that massive effort has been put in developing experimental tools combining the advantages (and avoiding as far as possible the drawbacks) of both approaches. Such tools, collectively designated as three-dimensional (3D) models, indeed provide a more natural culture environment, besides facilitating studies with the biological material of the relevant species, thus increasing the translational relevance of the study [1–3].

Current 3D models include a wide variety of approaches. In a simple form, primary cells or cell lines are guided towards 3D aggregates by providing a natural or synthetic scaffold. This basic principle, however, can be expanded to create advanced and biologically relevant multicellular spheroids [4]. Organoids, in contrast, are complex structures based on the long-term culture of stem cells or primary cells and ordered differentiation of their progeny under the influence of a cocktail of growth factors and/or chemicals in order to recapitulate the structural and functional properties of multiple adult organs [5, 6]. Organ-on-chip models integrate mechanical (such as shear or strain stress) and chemical (growth factors, cytokines) cues and include tailored sensing of the culture environment regarding aspects as medium flow rate, temperature, pH, partial pressure of gases, and mechanical forces, among many others [7–9]. Organ-on-chip are a particularly promising strategy for assessing the safety and efficacy of chemicals and pharmaceuticals [10]; the devices can harbor simple (cell lines) or complex (organoids) biological structures [11], and can be designed to allow communication between cell types of different organs, in what has been called multiorgan chips. Another recent advance is bioprinting, in which 3D printing-like techniques are used to combine cells, growth factors, and/or biomaterials to create 3D cell aggregates resembling tissues or organs [12, 13]. Due to ever improving cell culture conditions and chip manufacturing methods as well as a growing variety of printing techniques and an apparently unlimited availability of suitable biological and scaffold materials, the combination of 3D-based methods as organoids, organ-on-chip, tissue slices, and bioprinting (see Table 1 for a comparison of their main features), is likely to lastly bridge the gap between cell culture and a living organism [1, 14], significantly improving biomedical research and reducing the use of laboratory animals.

**Table 1:** Comparison of the key features of 3D systems

	Organoids	Organ-on-chip	Bioprinting	Tissue slices
Attainable complexity	Scalable, highly complex cellular composition by the combination of primary cells, stem cells and their progeny possible	Scalable, highly complex cellular composition and culture environments are possible	Highly defined and controlled assembling of cell types and matrices possible	Retain the original tissue architecture and complexity
Cell damage	Low or absent, but necrotic cores possible	Low or absent	Potentially high (temperature, shear stress)	Damage of adjacent cells unavoidable
Long-term culture	Virtually unlimited due to passaging (subcultivation)	Weeks to months, depending on the specific cell turnover and matrix properties	Depends on the specific cell turnover and matrix properties	Usually days to weeks
Non-preparative sampling	Supernatant, 3D imaging. Access to the apical surface may be difficult	Supernatants (compartment-wise, but limited volumes), sensor readouts; 3D imaging challenging	Supernatant, 3D imaging	Supernatant, 3D imaging
User-friendliness	Requires rather complex cell culture medium and additives	Sophisticated culture devices can be quite costly, time-consuming, and challenging to operate	Requires complex technologies, may be challenging in terms of operation and costs	Easy operation but requires recurrent tissue supply

## From Warburg’s shaving razor to precision-cut tissue slices

Tissue slices are a 3D model par excellence [14] and have been extensively used to address numerous research questions, including the study of intermediate metabolism in the liver or transport processes in the kidney. More recently, however, this method has been somewhat overshadowed by the general excitement around the organoid/organ-on-chip/bioprint trio [1–3]. Originally developed as an improvement of whole organ cultures, tissue slices have been in continuous use at least since the early 1900s, and thus decades before the establishment of the first cell line. Otto Warburg, for instance, generated tissue slices with a hand-guided razor blade for his Nobel prize-winning research on tumor metabolism [15]. Initially simple hand-held equipment, the devices improved over the decades to meet the need for uniform slice thickness, important for experiment reproducibility, and low sample waste when using small organs. A simple microtome for slice preparation from fresh tissue, described in 1944 [16], was followed by motor-driven [17] or hand-operated choppers [18], which reduced the time necessary for section preparation and did not require intensive training. A further improvement was the development of precision-cut tissue slices, namely the Krumdieck/Alabama [19] and later the Brendel/Vitron [20] slicers. With these apparatuses, which have been continuously improved regarding precision and ease of operation, slices are produced by mechanically moving an immobilized tissue cylinder across a microtome blade; the slice thickness can be adjusted within a rather large range of ~ 100 to 1000 µm, and tissue slicing is very rapid, with one slice produced every 3 to 4 s [21–23]. Both apparatuses seem to produce slices of comparable quality, as demonstrated by direct comparison of rat liver slices [24].

Slices should be cut at a thickness permitting efficient gas and metabolite exchanges; in most studies this ranges between 100 and 400 µm (see also Tables 2 and 3). Slices made too thick may show ischemic injury in the slice core, while slices made too thin may have a large proportion of damaged cells at their surface as compared to the total amount of healthy cells. Importantly, the thickness of precision-cut slices is much more constant, the number of damaged cells is greatly reduced, and the induction of immune responses is reduced compared to previous methods [25, 26]. Also, cultivation of such slices over prolonged periods (up to several weeks) has included different techniques, such as roller-tube cultures, culture on semipermeable membranes at the air–liquid interface, or embedding in 3D gels on culture dishes [27]. More recently, culture under continuous flow [28] or in microfluidic chambers [29] was reported.

**Table 2:** Selection of studies culturing vibrating microtome-generated slices from different organs or tissues

Organ/ tissue	Species	Slice thickness	Culture time (max)	Culture system features	Purpose	Ref
Brain	rt	300 µm	8 weeks	On insert, ALI, lentiviral infection	Protocol for creating hippocampal slices	[30]
	ms	110 µm	4 weeks	On insert, different Co-cultures	Protocol for co-cultures	[31]
	hu	250-350 µm	6 weeks	On insert, submerged,	Protocol for creating cortical slices	[32]
Spinal cord	rt	350 µm	14 days	On insert, ALI	Model development	[33]
Oculomotor nerve	ms	400-450 µm	72 h	On insert, submerged,	Study of oculomotor nerve outgrowth	[34]
Retina	ms, rt	40 – 170 µm	4 weeks	Submerged, in a LumiCycle	Study of circadian oscillations	[35]
	pg	250-300 µm	48 h	Within a gelatine sandwich, submerged	Morphometry and viability of photorecetors	[36]
	fs	150 µm	5 days	On coverslips, agarose coated	Method validation, interaction among retinal cells	[37]
	rt	125 µm	3 weeks	Plasma clot technique for cultivation	Electrophysiological recordings (patch clamp)	[38]
Olfactory epithelium	rt	400 µm	5 days	On coated inserts, submerged	Method for assessing olfactory development and function	[39]
Heart	rt, ms, hu, pg, dg	100-400 µm	7 days	ALI	Model development	[40]
	hu, ms	380 µm	4 days	In chip, dynamic, with sensors	Shippable model for pre-clinical drug testing and basic research	[41]
	hu, pg	300 µm	6 days	Submerged, electrical stimulation, media oxygenation	Model for drug testing and gene therapy	[42]
	ms	300 µm	6 days	On insert, ALI	Model for gene therapy, gene transfer efficiency, cell tropism, and toxicity	[43]
	hu	400 µm	48 h	On insert, ALI	SARS-CoV-2 infection model	[44]
	hu	300 µm	14 days	Submerged, application of pre- and afterload	Analysis of contraction force and kinetics	[45]
	pg	400-500 µm	48 h	On PDMS pillars, ALI, with/without insert, static and dynamic	Study epicardial cell physiology and activation	[46]
Lung	ms	275 µm	5 days	On insert, ALI	Model for circadian timing in lung, role of Clara cells and glucocorticoids	[47]
	hu, ms	500 µm, 300 µm	5 days	Submerged, rolling, co-culture with transfected fibroblasts	Multidimensional immunolabeling and 4D time-lapse imaging of vital slices	[48]
	hu, ms	500 µm, 300 µm	14 days	Submerged	Wnt-induced repair, 4D confocal live tissue imaging	[49]
	ms	150 µm	15 days	Submerged	Study of small airway smooth muscle contraction	[50]
	rt	500 µm	12 h	Stretcher to mimic breathing mechanic	Analysis of response to cigarette smoke	[51]
	pg, hm, ct	350 µm	4 days	Submerged	Check for susceptibility for SARS-CoV-2	[52]
	ms	300 µm	4 days	Submerged	Study of pulmonary fibrosis disease mechanisms	[53]
Salivary gland	hu	35 µm and 50 µm	14 days	On insert, ALI	Slice culture model development	[54]
	ms	50 µm	2 days	Submerged	Slice culture model development with emphasis on imaging	[55]
Small Intestine	ms	250 µm	6 days	Slices covered with collagen and medium on top	Model for interactions of different cell types in the intestine	[56]
	ms	250 µm	48 h	Slices covered with collagen and medium on top	Neuronal regulation of goblet cell production by	[57]
Colon	hu	250 µm	3 days	On collagen-covered slices, submerged, co-culture with *S. typhimurium*	Host microbial interactions, influence of oxygen availability	[58]
Prostate	hu	200-300 µm	10 days	Submerged, hypoxia	Prostate tissue model	[59]
Pancreas	ms	100 µm	12 weeks	On insert, ALI; electrophysiology	β-cell physiology modell establishment	[60]
Liver	hu, rt	100-400 µm	28 days	On insert, ALI	Fibrosis model	[61]
	ms	100-250 µm	5 days	Submerged	Model for chronic liver diseases	[62]
	ms, hu	250 µm	5 days	Submerged	Improve slice culture, fibrosis drug therapy testing	[63]
Spleen	ms	230 µm	4 days	Submerged	Protocol, method validation	[64]
Thymus	ms	400–500 µm	Several days	On insert, submerged; overlayed with thymocyte cell suspension	Model for studying T cell development	[65]
Femur	rt	300-400 µm	3 weeks	On insert	Study enchondral osteogenesis	[66]
Meibomian gland	ms	150 µm	21 days	Submerged	Model development, effect of melanocortins on secretion	[67]
Endometrium	hu	200 µm	48 h	Submerged	Modulation of endometrial PGE2 synthesis	[68]

*ALI *Air–liquid interface, *hu *human, *rt *rat, *ms *mouse, *pg *pig, *dg *dog, *hm *hamster, *ct *cat

**Table 3:** Non-exhaustive selection of studies culturing vibrating microtome-generated slices from different tumor types

Tumor type	Species	Slice thickness	Culture time (max)	Culture system features	Purpose	Ref
Lung	hu	300 µm	6 months	Implanted into mice (xenograft)	Model for primary tumor expansion and xenograft production	[69]
	sh, ms	300 µm	1 month	Submerged	Standardized slice model for viral infection gene therapy	[70]
	ms	160-250 µm	3 days	ALI, titanium grid, rotation	Tumor drug testing model	[71]
Oral squamous cell carcinoma	hu	350–450 µm	8 days	Chorioallantoic membrane (CAM)	Establishing slices on CAM as a tumor model	[72]
Gastrointestinal (various)	hu	250 µm	7 days	On insert, submerged	Protocol, method evaluation	[73]
Prostate	hu	200-300 µm	10 days	Submerged, hypoxia	Prostate tumor model	[59]
	ms	300 µm	6 days	With/without insert and strainer	Establishing a chemotherapy model	[74]
	hu	250 µm	9 days	On insert, ALI	Model for immune microenvironment studies	[75]
	hu	350 µm	96 h	On insert, submerged	Model development	[76]
	hu	350 µm	96 h	On insert, submerged	Assessing culture effects by transcriptome profiling	[77]
	hu	250 µm	9 days	On insert, ALI	Model development	[78]
	hu	250 µm	4 days	On insert, ALI	Tumor immunology studies	[79]
	hu	300 µm	15 days	On insert, ALI	Method development	[80]
	hu	250 µm	6 days	On insert, submerged	Interaction of tumor cells with immune microenvironment	[81]
Liver	hu	200-300 µm	3 days	Submerged	Comparison of slicing devices	[82]
	hu, ms	200-300 µm	7 days	On insert, submerged	Drug discovery, immuno-oncology	[83]
	hu	250 µm	4 days	On insert, submerged	Immune checkpoint ligands and chemotherapy response	[84]
	hu	250 µm	6 days	On insert, submerged	Establish CarT-cell treatment model	[85]
	hu	250 µm	3 days	On insert, submerged	Neutralizing antibodies and CAR-T cells in cancer therapy	[86]
Bladder	hu	300 µm	2 days	On insert, submerged, on a rotating plate	Method for studying oncolytic viruses	[87]
Kidney	hu	300 µm	1 week	Submerged	Characterization of the tumor immune environment	[88]
Uterine leiomyoma	hu	500 µm	3 weeks	On alginate scaffold discs	Model development	[89]
Breast	hu	300 µm	7 days	Submerged with/without rotating platform	Model development, comparison with manual slicing	[90]
	hu	250 µm	7 days	Submerged	Model evaluation	[91]
	hu	250 µm	3 days	Submerged	Establishing a chemotherapy model	[92]
Breast (xenograft)	hu	200 µm	4 days	Submerged	Drug testing	[93]
Breast and prostate PDX models	hu	300 µm	2 weeks (breast), 1 week (prostate)	In chip, submerged, shear stress, perfusion	Cancer on chip platform for predicting drug response	[94]
Head and Neck Squamous Cell Carcinoma	hu	300 µm	5 days	Rotating platform	Model for evaluation of treatment response to radiation and chemotherapy	[95]

*ALI* Air–liquid interface, *PDX* Patient-derived xenograft, *hu* Human, *ms* mouse, *sh* sheep

Several features contributed to a high popularity of “precision-cut slices”, including: a) the ECM and all or at least most supportive cell types are already present in the correct tissue architecture; b) all cell types are isogenic; c) there is no enzymatic dissociation, thus preserving cell surface proteins (however, tissue damage during slicing can induce immune responses); d) it is amenable for imaging; e) a large number of slices can usually be obtained from a single organ; f) it is an accessible, unexpensive and technically rather simple procedure. Therefore, slice production and culture methods were next extensively improved and adapted to different requirements and became an essential part of the toolbox in most fields of biomedical research, including neuroscience [96], lung [97] and liver [98] diseases, and host–pathogen interactions [99].

## Vibrating microtomes enter the stage

**Fig. 1:**
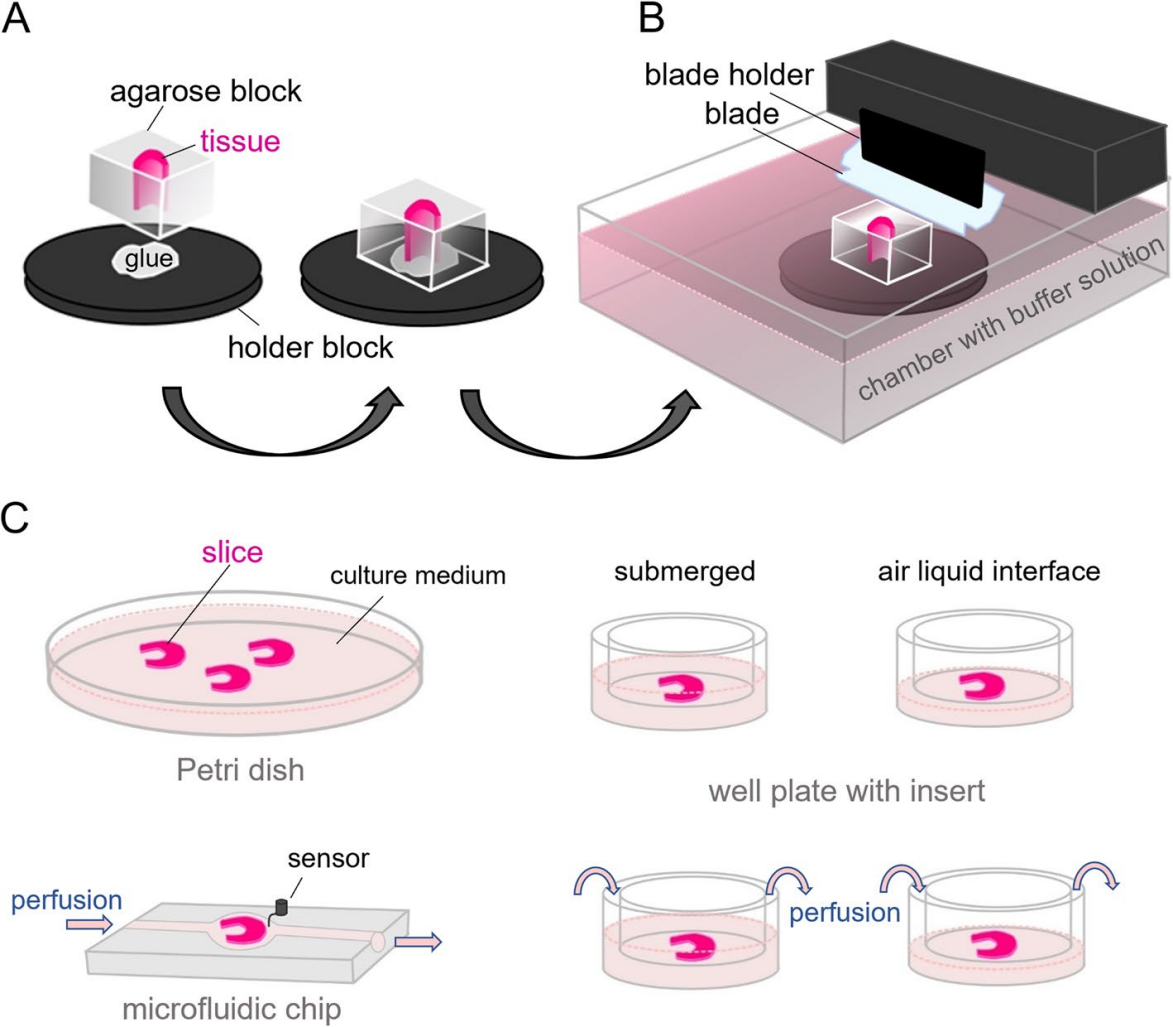
Working principle of tissue slice culture. **A** The sample is embedded in agarose and sticked to the block holder (left). **B** Side-view of a typical vibratome cutting chamber (right). **C** Typical culture methods employed downstream of slice generation: submerged in culture medium, on insert submerged or at the air–liquid interface, in each case with or without perfusion, and in a microfluidic culture chamber. Figures were created using Krita (https://krita.org)

In a recent survey, Dewyse and colleagues [98] report that while the majority of precision-cut liver slices is still generated with the Krumdieck or Brendel slicers, vibrating blade microtomes are gaining popularity. These devices were originally developed at Oxford Laboratories in California [100] and later marketed by different companies including Leica, currently the owner of the brand name “vibratome”. The hallmark of the apparatus is the lateral, saw-like movement of the blade as it progresses, which significantly reduces tissue pulling or tearing compared to a straight cut (Table 4). In a typical setup, fresh tissue samples (either tissue pieces or punch-generated tissue cores) are embedded in low gelling temperature agarose blocks (Fig. 1A) and attached with contact glue to a holder within the cutting chamber of the vibratome (Fig. 1B). The holder is raised or lowered to adjust the thickness of the section as a sharp blade moves and cuts in a plane parallel to the sample’s surface. During cutting, both the sample and the blade edge are immersed in an aqueous buffer, resulting in the formation of free-floating sections, which can be immediately imaged, fixed and histologically processed, enzymatically dissociated for obtaining individual cell populations, or employed for cultivation and manipulation in vitro.

**Table 4:** Cutting by pressing and sliding: lessons from cheese and salami slicing

Cutting of soft materials, be it human flesh by the surgeon, meat or vegetables by the chef, or tissue samples by the histologists, is made considerably easier by sliding the blade rather than just pressing it against the surface of the object to be cut. This principle also holds for the common “paper cut”, the painful rendezvous between the skin and a thin paper sheet. The phenomenon has been modelled with a wide variety of materials, and different explanations have been provided. Atkins and colleagues [101] lively review the mechanics of cutting and define our study object as a material in which sectioning creates a floppy offcut that is not permanently deformed and has negligible bending resistance. These authors demonstrate (by cutting cheddar cheese and salami) that the greater the “slice/push ration”, the lower the necessary cutting forces. Reyssat and colleagues [102] focus on the role played by shear forces on gelatin blocks and reveal that the sliding action creates a critical local tension at the contact site, in contrast to a strong global tension caused by pressing only. Thus, under slicing (= blade vibration) conditions, there is less global deformation and material damage, resulting in better preserved slices – in our context the key motivation for employing vibrational cutting	

Finding the suitable vibratome settings for the tissue of interest and establishing a protocol for an optimal slicing procedure can be time-consuming. Factors to be considered are the rigidity or elasticity of the tissue itself, the type of supportive embedding material, and the settings of the instrument, including the desired slice thickness, the angle and amplitude of the blade, and its propulsion speed. The downstream processing of the slices also needs to be considered, as the dehydration associated to sample fixation can lead to shrinkage in slice thickness of over 50% [103]. While in principle any tissue can be sliced with this technique, samples that are very soft, contain hard components, or are rich in elastic elements are less suitable than homogeneous samples and may require extensive protocol improvement. Although some studies focused on the impact of specific device settings and the type of sample (fresh vs. fixed) on slice properties [104], or on the impact of the viscoelastic properties of the embedding structure on slice viability and quality [105], the diversity of sample types makes it difficult to provide exact recommendations regarding the device settings. As a general rule, soft and elastic tissues should be cut at higher amplitude and lower mechanic deflection and propulsion than more rigid tissue types. Researchers may initially apply the settings used in previous publications employing the same tissue (see Tables 2 and 3 for numerous examples), but optimal slicing conditions frequently need to be established empirically.

By directly comparing a vibratome with a Krumdieck tissue slicer, Zimmermann and colleagues [82] showed that the vibratome, while requiring longer operation time, is superior in terms of accuracy and reproducibility. However, the technique can also be applied in fixed or cleared tissue samples, and has been integrated into numerous imaging platforms [106, 107]. Importantly, the technical features of the apparatuses are being constantly improved. For example, Wang and colleagues determined that the sectioning quality of soft materials can be enhanced by using higher sectioning frequency, blade oscillation amplitude, and lower sample feed rate [108]. As a result, the same group developed a novel high-frequency vibrating microtome allowing high-speed cutting without compromising slice quality, and successfully applied it to organ-wide imaging [109].

As a gentle cutting technique, it is particularly suitable for creating fresh tissue slices to be further cultured. Upon identification of appropriate sample thickness and other cutting parameters, optimal culture conditions can be established. Tissue slices generated with a vibrating microtome have been cultured under a large variety of conditions: simply submerged in culture medium, on membrane-coated inserts, or at the air–liquid interface, in each case with or without perfusion, or in microfluidic devices, among others (Fig. 1C). Such systems have been widely used for housing and manipulating tissue slices for a variety of purposes and experimental questions. A non-exhaustive overview of investigations involving the culture of vibrating microtome-derived normal and tumor tissues is given in Tables 2 and 3, respectively.

## Perspectives and conclusions

Vibrating microtome sectioning is an outstanding tool for creating tissue slices suitable for a wide variety of studies and amenable for numerous cutting-edge imaging technologies [14, 110]. Latest downstream applications include tissue regeneration studies [53, 63] or the evaluation of cancer treatment strategies like gene therapy, where the transfection efficacy in a complex environment can be assessed [43]. Furthermore, immunotherapy precision and the invasion of Car T-cells can be tracked [86]. As immune cells are present in a natural architecture within the slices, they are a suitable model for the investigation of host–pathogen interactions [52, 99, 111], host- microbiome interactions [58] and drug safety assessment [42, 71]. Nevertheless, it should not be omitted that this method also comes with several potential drawbacks, including the increased appearance of apoptotic or necrotic areas directly at or close to areas damaged by the blade (including anoikis induced by ECM removal), disruption or clogging of vessels, depletion of specific cell types (for instance by migration into the medium), and considerably reduced supply and removal of substrates and metabolites compared to the uncut tissue. These problems may represent a significant challenge and require considerable improvement of tissue slice culture techniques.

Overall, this strategy will benefit from diverse technical improvements and subsequent developments.

### Vibrating microtome technical improvements

While vibrating microtomes are easy to use and, compared to standard tissue choppers, more sample-gently, slice preparation is more time-consuming. The time needed for sample processing may indeed be especially critical for enzyme-rich (pancreas) or highly metabolic (liver) organs. To overcome this issue, e.g. for liver samples, sophisticated media can be combined with low temperatures during slicing, thus improving slice quality and viability [63]. Therefore, an automated temperature control of sample holder and media would be favorable, as for the most systems manual addition of crushed ice remains necessary. During sample preparation, the rigidity of relatively soft and flexible tissue types like skin, intestine, or lung needs to be increased. While appropriate stabilizing agents like low melting point agaroses are available, getting the slices completely rid of their remnants remains an issue. During sequential sample processing, usually slices end up floating around in the sample chamber filled with media, while the blade is already beginning to move to generate the next slice. Here, the inclusion of a medium stream gadget in the instruments to transport the floating slices away from the blade, thereby preventing sample damage and facilitating slice transfer to culture systems, would be desirable. Although most operators work with antibiotics in the media, it may be favorable to work without these additives for some applications. As the footprint of some devices is rather small, sterile working conditions can be readily achieved by placing the vibrating microtome under a sterile hood. In contrast, there is currently no vibrating microtome available containing a self-sterilization function or including a sterile working chamber. Finally, as for the regular microtomes, working safety is a critical issue and accidents may occur. Therefore, a corresponding cap to cover the razor blade when not in use, as well as an easily accessible emergency stop button would be useful additions.

### Combination with other 3D-models and downstream applications

3D models shouldn’t be seen as stand-alone techniques, as only the combination of different approaches may result in a physiologically relevant model. Tissue slices combined with organ-on-chip technology enable sensor implementation into the culture device and a tight control of parameters [41]. This enables sophisticated manipulation of culture conditions and thereby mimic homeostatic or dysbiotic conditions. Organ-on-chip/microfluidics systems may also allow reproducing one of the key properties of tissues in vivo, the continuous nutritional supply, gas exchange, and removal or transport of metabolites and growth factors via capillarization. These processes maintain important biophysiochemical gradients alongside the endothelial-epithelial axis, and its implementation in in vitro models is essential for improving the translational value of the studies. Furthermore, certain cell types may require perfusion as they respond towards the corresponding shear stress with an increased barrier function and morphological adaptions [112]. Especially in case of linear perfusion, microfluidic devices can help to decrease the amount of media consumption. With regard to specific applications, dynamic cultivation used in infection experiments might contribute to prevent microbial overgrowth [113] or provide more in vivo-like infection conditions, for instance when studying invasion mechanisms [114].

From a practical point of view, as most tissue slices are cultured on inserts, perfusable plates are one option for dynamic cultivation conditions, and the same applies to chip-systems containing a porous membrane as separator for perfusable compartments. In such systems, one compartment contains the tissue slice, and the other one can be perfused. Depending on the tissue type, site-specific, ubiquitous, unidirectional, or bidirectional perfusion can be applied. However, perfusion similar to that in vivo is difficult to achieve in microfluidic chambers, as vessel anastomosis in bioreactors is usually missing. Also, the multicellular tissue slices are exposed to a single media type, and a site-specific application of shear stress can hardly be realized. This could negatively affect cell viability and function of tissues not exposed to shear stress under physiological conditions (for instance, interstitial tissue).

Co-culture of slices with cell lines opens up a broad spectrum of investigations. For instance, fibroblasts added to preliminary injured tissue slices can be used for the investigation of fibrosis mechanisms [48]. In another study, Car T-cell and genetically engineered macrophage invasion in tumor tissue slices were examined [85]). Not only mammalian cells, but also pathogens as SARS-CoV-2 have been co-cultured with tissue slices in order to determine cell type susceptibility for the virus and thereby identify potential treatment targets [44, 52].

Tissue slices can be combined with animal models as well. Frequently, the initial manipulation takes place in vivo, and the subsequent generation of tissue slices greatly amplifies the number of samples available for further in vitro treatments. In this way, the number of animals used in experiments can be reduced. However, the experimental setup can also be designed inversely, as tissue slices of one species can be implanted into another one in the form of xenografts [69].

### Sample analysis and logistics

Co-evolving imaging technologies of live tissue imaging (4D) enable whole sample analysis, time-lapse recording of viable tissue slices enabling thereby e.g., in-tissue observation of cell migration and tissue regeneration [49]. The analysis of living and fixed tissue slices via cLSM and light-sheet microscopy permits a 3D-reconstruction of native and manipulated tissue in all its complexity [30, 55]. Of course, preparative downstream analysis of vital tissue, including single cell analysis and studies on ECM function, production and regeneration, so far mainly performed with tissue chopper-generated slices, can be carried out on vibrating microtome-generated slices as well [115–117]. Similarly to slides generated with standard microtomes, vibrating microtome slices would significantly benefit from cryopreservation methods, as hundreds or thousands of slices may be created from a single organ, cryopreserved, and used on demand [118]. Notably, test platforms combining precision-cut slices with cryopreservation for assessing drug response of hepatic tumors [119] or assessing the immune response of the lung [120] have been recently reported.

To conclude, there is great potential for the combination of vibrating microtome tissue slices with microfluidic culture devices, which have been greatly improved in the context of organ-on-chip methods regarding the modulation of specific culture conditions and the use of miniaturized sensors. In particular, the correct tissue-like spatial organization, multicellularity and the presence of native ECM of such slices in combination with tightly controlled culture conditions will provide a unique model for assessing organ physiology and testing the effects of substances in vivo.

## Data Availability

Not applicable.

## Abbreviations

3D: Three-dimensional
ECM: Extracellular matrix

## References

[1] Picollet-D'hahan N, Dolega ME, Liguori L, Marquette C, Le Gac S, Gidrol X, Martin DK (2016). A 3D Toolbox to Enhance Physiological Relevance of Human Tissue Models. Trends Biotechnol.

[2] Jackson EL, Lu H (2016). Three-dimensional models for studying development and disease: moving on from organisms to organs-on-a-chip and organoids. Integr Biol (Camb).

[3] Cacciamali A, Villa R, Dotti S (2022). 3D Cell Cultures: Evolution of an Ancient Tool for New Applications. Front Physiol..

[4] van Os EA, Cools L, Eysackers N, Szafranska K, Smout A, Verhulst S (2022). Modelling fatty liver disease with mouse liver-derived multicellular spheroids. Biomaterials..

[5] Turner DA, Baillie-Johnson P, Martinez AA (2016). Organoids and the genetically encoded self-assembly of embryonic stem cells. BioEssays.

[6] Sato T, Clevers H (2015). SnapShot: Growing Organoids from Stem Cells. Cell.

[7] Bhatia SN, Ingber DE (2014). Microfluidic organs-on-chips. Nat Biotechnol.

[8] Kavand H, Nasiri R, Herland A (2022). Advanced Materials and Sensors for Microphysiological Systems: Focus on Electronic and Electrooptical Interfaces. Adv Mater..

[9] Mou L, Mandal K, Mecwan MM, Hernandez AL, Maity S, Sharma S (2022). Integrated biosensors for monitoring microphysiological systems. Lab Chip.

[10] Schneider MR, Oelgeschlaeger M, Burgdorf T, van Meer P, Theunissen P, Kienhuis AS (2021). Applicability of organ-on-chip systems in toxicology and pharmacology. Crit Rev Toxicol.

[11] Park SE, Georgescu A, Huh D (2019). Organoids-on-a-chip Science.

[12] Deo KA, Singh KA, Peak CW, Alge DL, Gaharwar AK (2020). Bioprinting 101: Design, Fabrication, and Evaluation of Cell-Laden 3D Bioprinted Scaffolds. Tissue Eng Part A.

[13] Ramesh S, Harrysson OL, Rao PK, Tamayol A, Cormier DR, Zhang Y, Rivero IV (2021). Extrusion bioprinting: Recent progress, challenges, and future opportunities. Bioprinting..

[14] Pampaloni F, Reynaud EG, Stelzer EHK (2007). The third dimension bridges the gap between cell culture and live tissue. Nat Rev Mol Cell Biol.

[15] Warburg O. Versuche an Überlebendem Karcinomgewebe. Biochemische Zeitschrift. 1923:317–33.

[16] Stadie WC, Riggs BC (1944). MICROTOME FOR THE PREPARATION OF TISSUE SLICES FOR METABOLIC STUDIES OF SURVIVING TISSUES IN VITRO. J Biol Chem.

[17] McIlwain H, BuddlE HL. Techniques in tissue metabolism. I. A mechanical chopper. Biochem J. 1953;53:412–20. doi:10.1042/bj0530412. 10.1042/bj0530412 PMC1198164 13032086

[18] Mahler DJ, Humoller FL (1965). Tissue chopper for biochemical studies. Anal Biochem.

[19] Krumdieck CL, dos Santos JE, Ho KJ (1980). A new instrument for the rapid preparation of tissue slices. Anal Biochem.

[20] Brendel K, Fisher RL, Krumdieck CL, Gandolfi AJ (1990). Precision-Cut Rat Liver Slices in Dynamic Organ Culture for Structure-Toxicity Studies. J Am Coll Toxicol.

[21] Parrish AR, Gandolfi AJ, Brendel K (1995). Precision-cut tissue slices: applications in pharmacology and toxicology. Life Sci.

[22] Lerche-Langrand C, Toutain HJ (2000). Precision-cut liver slices: characteristics and use for in vitro pharmaco-toxicology. Toxicology.

[23] de Graaf IAM, Olinga P, de Jager MH, Merema MT, de Kanter R, van de Kerkhof EG, Groothuis GMM (2010). Preparation and incubation of precision-cut liver and intestinal slices for application in drug metabolism and toxicity studies. Nat Protoc.

[24] Price RJ, Ball SE, Renwick AB, Barton PT, Beamand JA, Lake BG (1998). Use of precision-cut rat liver slices for studies of xenobiotic metabolism and toxicity: comparison of the Krumdieck and Brendel tissue slicers. Xenobiotica.

[25] de Kanter R, Monshouwer M, Meijer DKF, Groothuis GMM (2002). Precision-cut organ slices as a tool to study toxicity and metabolism of xenobiotics with special reference to non-hepatic tissues. Curr Drug Metab.

[26] Henjakovic M, Sewald K, Switalla S, Kaiser D, Müller M, Veres TZ (2008). Ex vivo testing of immune responses in precision-cut lung slices. Toxicol Appl Pharmacol.

[27] Gähwiler BH, Capogna M, Debanne D, McKinney RA, Thompson SM (1997). Organotypic slice cultures: a technique has come of age. Trends Neurosci.

[28] Schumacher K, Khong Y-M, Chang S, Ni J, Sun W, Yu H (2007). rfusion culture improves the maintenance of cultured liver tissue slices. Tissue Eng.

[29] van Midwoud PM, Groothuis GMM, Merema MT, Verpoorte E (2010). Microfluidic biochip for the perifusion of precision-cut rat liver slices for metabolism and toxicology studies. Biotechnol Bioeng.

[30] Church TW, Gold MG (2021). Preparation of Rat Organotypic Hippocampal Slice Cultures Using the Membrane-Interface Method. Methods Mol Biol.

[31] Humpel C (2018). Organotypic Brain Slice Cultures. Curr Protoc Immunol..

[32] Kvist G (2021). Derivation of Adult Human Cortical Organotypic Slice Cultures for Coculture with Reprogrammed Neuronal Cells. Methods Mol Biol.

[33] Liu J-J, Huang Y-J, Xiang L, Zhao F, Huang S-L (2017). A novel method of organotypic spinal cord slice culture in rats. NeuroReport.

[34] Whitman MC, Bell JL, Nguyen EH, Engle EC (2019). Ex Vivo Oculomotor Slice Culture from Embryonic GFP-Expressing Mice for Time-Lapse Imaging of Oculomotor Nerve Outgrowth. J Vis Exp.

[35] Jaeger C, Sandu C, Malan A, Mellac K, Hicks D, Felder-Schmittbuhl M-P (2015). Circadian organization of the rodent retina involves strongly coupled, layer-specific oscillators. FASEB J.

[36] Khodair MA, Zarbin MA, Townes-Anderson E (2005). Cyclic AMP prevents retraction of axon terminals in photoreceptors prepared for transplantation: an in vitro study. Invest Ophthalmol Vis Sci.

[37] Mack AF, Fernald RD (1991). Thin slices of teleost retina continue to grow in culture. J Neurosci Methods.

[38] Feigenspan A, Bormann J (1994). Modulation of GABAC receptors in rat retinal bipolar cells by protein kinase C. J Physiol.

[39] Gong Q, Liu WL, Srodon M, Foster TD, Shipley MT (1996). Olfactory epithelial organotypic slice cultures: a useful tool for investigating olfactory neural development. Int J Dev Neurosci.

[40] Watson SA, Scigliano M, Bardi I, Ascione R, Terracciano CM, Perbellini F (2017). Preparation of viable adult ventricular myocardial slices from large and small mammals. Nat Protoc.

[41] Qiao Y, Dong Q, Li B, Obaid S, Miccile C, Yin RT (2019). Multiparametric slice culture platform for the investigation of human cardiac tissue physiology. Prog Biophys Mol Biol.

[42] Ou Q, Jacobson Z, Abouleisa RRE, Tang X-L, Hindi SM, Kumar A (2019). Physiological Biomimetic Culture System for Pig and Human Heart Slices. Circ Res.

[43] Liu Z, Klose K, Neuber S, Jiang M, Gossen M, Stamm C (2020). Comparative analysis of adeno-associated virus serotypes for gene transfer in organotypic heart slices. J Transl Med.

[44] Brumback BD, Dmytrenko O, Robinson AN, Bailey AL, Ma P, Liu J (2022). Human Cardiac Pericytes are Susceptible to SARS-CoV-2 Infection. JACC Basic Transl Sci.

[45] Hamers J, Sen P, Merkus D, Seidel T, Lu K, Dendorfer A (2022). Preparation of Human Myocardial Tissue for Long-Term Cultivation. J Vis Exp.

[46] Maselli D, Matos RS, Johnson RD, Chiappini C, Camelliti P, Campagnolo P (2022). Epicardial slices: an innovative 3D organotypic model to study epicardial cell physiology and activation. NPJ Regen Med.

[47] Gibbs JE, Beesley S, Plumb J, Singh D, Farrow S, Ray DW, Loudon ASI (2009). Circadian timing in the lung; a specific role for bronchiolar epithelial cells. Endocrinology.

[48] Burgstaller G, Vierkotten S, Lindner M, Königshoff M, Eickelberg O (2015). Multidimensional immunolabeling and 4D time-lapse imaging of vital ex vivo lung tissue. Am J Physiol Lung Cell Mol Physiol.

[49] Uhl FE, Vierkotten S, Wagner DE, Burgstaller G, Costa R, Koch I (2015). Preclinical validation and imaging of Wnt-induced repair in human 3D lung tissue cultures. Eur Respir J.

[50] Li G, Cohen JA, Martines C, Ram-Mohan S, Brain JD, Krishnan R (2020). Preserving Airway Smooth Muscle Contraction in Precision-Cut Lung Slices. Sci Rep.

[51] Mondoñedo JR, Bartolák-Suki E, Bou Jawde S, Nelson K, Cao K, Sonnenberg A (2020). A High-Throughput System for Cyclic Stretching of Precision-Cut Lung Slices During Acute Cigarette Smoke Extract Exposure. Front Physiol.

[52] Gerhards NM, Cornelissen JBWJ, van Keulen LJM, Harders-Westerveen J, Vloet R, Smid B (2021). Predictive Value of Precision-Cut Lung Slices for the Susceptibility of Three Animal Species for SARS-CoV-2 and Validation in a Refined Hamster Model. Pathogens.

[53] Stancil IT, Michalski JE, Hennessy CE, Hatakka KL, Yang IV, Kurche JS, et al. Interleukin-6-dependent epithelial fluidization initiates fibrotic lung remodeling. Sci Transl Med. 2022;14:eabo5254. doi:10.1126/scitranslmed.abo5254. 10.1126/scitranslmed.abo5254 PMC9981332 35857823

[54] Su X, Fang D, Liu Y, Ramamoorthi M, Zeitouni A, Chen W, Tran SD (2016). Three-dimensional organotypic culture of human salivary glands: the slice culture model. Oral Dis.

[55] Warner JD, Peters CG, Saunders R, Won JH, Betzenhauser MJ, Gunning WT (2008). Visualizing form and function in organotypic slices of the adult mouse parotid gland. Am J Physiol Gastrointest Liver Physiol.

[56] Schwerdtfeger LA, Ryan EP, Tobet SA (2016). An organotypic slice model for ex vivo study of neural, immune, and microbial interactions of mouse intestine. Am J Physiol Gastrointest Liver Physiol.

[57] Schwerdtfeger LA, Tobet SA (2020). Vasoactive intestinal peptide regulates ileal goblet cell production in mice. Physiol Rep..

[58] Schwerdtfeger LA, Nealon NJ, Ryan EP, Tobet SA (2019). Human colon function ex vivo: Dependence on oxygen and sensitivity to antibiotic. PLoS One..

[59] Figiel S, Pasqualin C, Bery F, Maupoil V, Vandier C, Potier-Cartereau M (2019). Functional Organotypic Cultures of Prostate Tissues: A Relevant Preclinical Model that Preserves Hypoxia Sensitivity and Calcium Signaling. Am J Pathol.

[60] Meneghel-Rozzo T, Rozzo A, Poppi L, Rupnik M (2004). In vivo and in vitro development of mouse pancreatic beta-cells in organotypic slices. Cell Tissue Res.

[61] Verrill C, Davies J, Millward-Sadler H, Sundstrom L, Sheron N (2002). Organotypic liver culture in a fluid-air interface using slices of neonatal rat and adult human tissue–a model of fibrosis in vitro. J Pharmacol Toxicol Methods.

[62] Pearen MA, Lim HK, Gratte FD, Fernandez-Rojo MA, Nawaratna SK, Gobert GN (2020). Murine Precision-Cut Liver Slices as an Ex Vivo Model of Liver Biology. J Vis Exp.

[63] Dewyse L, de Smet V, Verhulst S, Eysackers N, Kunda R, Messaoudi N (2022). Improved Precision-Cut Liver Slice Cultures for Testing Drug-Induced Liver Fibrosis. Front Med (Lausanne)..

[64] Finetti F, Capitani N, Manganaro N, Tatangelo V, Libonati F, Panattoni G (2020). Optimization of Organotypic Cultures of Mouse Spleen for Staining and Functional Assays. Front Immunol.

[65] Ross JO, Melichar HJ, Halkias J, Robey EA (2016). Studying T Cell Development in Thymic Slices. Methods Mol Biol.

[66] Srinivasaiah S, Musumeci G, Mohan T, Castrogiovanni P, Absenger-Novak M, Zefferer U (2019). A 300 μm Organotypic Bone Slice Culture Model for Temporal Investigation of Endochondral Osteogenesis. Tissue Eng Part C Methods.

[67] Zahn I, Garreis F, Schicht M, Rötzer V, Waschke J, Liu Y (2022). A New Organotypic 3D Slice Culture of Mouse Meibomian Glands Reveals Impact of Melanocortins. Int J Mol Sci.

[68] Tabibzadeh S, Kaffka KL, Satyaswaroop PG, Kilian PL (1990). Interleukin-1 (IL-1) regulation of human endometrial function: presence of IL-1 receptor correlates with IL-1-stimulated prostaglandin E2 production. J Clin Endocrinol Metab.

[69] Russo MV, Faversani A, Gatti S, Ricca D, Del Gobbo A, Ferrero S (2015). A new mouse avatar model of non-small cell lung cancer. Front Oncol.

[70] Rosales Gerpe MC, van Vloten JP, Santry LA, de Jong J, Mould RC, Pelin A (2018). Use of Precision-Cut Lung Slices as an Ex Vivo Tool for Evaluating Viruses and Viral Vectors for Gene and Oncolytic Therapy. Mol Ther Methods Clin Dev.

[71] Nagaraj AS, Bao J, Hemmes A, Machado M, Närhi K, Verschuren EW (2018). Establishment and Analysis of Tumor Slice Explants As a Prerequisite for Diagnostic Testing. J Vis Exp.

[72] Kauffmann P, Troeltzsch M, Brockmeyer P, Bohnenberger H, Heidekrüger PI, Manzke M (2018). First experience of chick chorioallantoic membrane (CAM) assay in the clinical work flow with oral squamous cell carcinoma patients. Clin Hemorheol Microcirc.

[73] Kenerson HL, Sullivan KM, Seo YD, Stadeli KM, Ussakli C, Yan X (2020). Tumor slice culture as a biologic surrogate of human cancer. Ann Transl Med.

[74] Zhang W, van Weerden WM, de Ridder CMA, Erkens-Schulze S, Schönfeld E, Meijer TG (2019). Ex vivo treatment of prostate tumor tissue recapitulates in vivo therapy response. Prostate.

[75] Jiang X, Seo YD, Chang JH, Coveler A, Nigjeh EN, Pan S (2017). Long-lived pancreatic ductal adenocarcinoma slice cultures enable precise study of the immune microenvironment. Oncoimmunology..

[76] Misra S, Moro CF, Del Chiaro M, Pouso S, Sebestyén A, Löhr M (2019). Ex vivo organotypic culture system of precision-cut slices of human pancreatic ductal adenocarcinoma. Sci Rep.

[77] Ghaderi M, Fernández Moro C, Pouso Elduayen S, Hultin E, Verbeke CS, Björnstedt M, Dillner J (2020). Genome-wide transcriptome profiling of ex-vivo precision-cut slices from human pancreatic ductal adenocarcinoma. Sci Rep.

[78] Lim CY, Chang JH, Lee WS, Lee KM, Yoon YC, Kim J, Park IY (2018). Organotypic slice cultures of pancreatic ductal adenocarcinoma preserve the tumor microenvironment and provide a platform for drug response. Pancreatology.

[79] Lim CY, Chang JH, Lee WS, Kim J, Park IY (2022). CD40 Agonists Alter the Pancreatic Cancer Microenvironment by Shifting the Macrophage Phenotype toward M1 and Suppress Human Pancreatic Cancer in Organotypic Slice Cultures. Gut Liver.

[80] Braun R, Lapshyna O, Eckelmann S, Honselmann K, Bolm L, ten Winkel M (2021). Organotypic Slice Cultures as Preclinical Models of Tumor Microenvironment in Primary Pancreatic Cancer and Metastasis. J Vis Exp.

[81] Jiang X, Seo YD, Sullivan KM, Pillarisetty VG (2019). Establishment of Slice Cultures as a Tool to Study the Cancer Immune Microenvironment. Methods Mol Biol.

[82] Zimmermann M, Lampe J, Lange S, Smirnow I, Königsrainer A, Hann-von-Weyhern C (2009). Improved reproducibility in preparing precision-cut liver tissue slices. Cytotechnology.

[83] Sivakumar R, Chan M, Shin JS, Nishida-Aoki N, Kenerson HL, Elemento O (2019). Organotypic tumor slice cultures provide a versatile platform for immuno-oncology and drug discovery. Oncoimmunology..

[84] Jabbari N, Kenerson HL, Lausted C, Yan X, Meng C, Sullivan KM (2020). Modulation of Immune Checkpoints by Chemotherapy in Human Colorectal Liver Metastases. Cell Rep Med..

[85] Kenerson HL, Sullivan KM, Labadie KP, Pillarisetty VG, Yeung RS (2021). Protocol for tissue slice cultures from human solid tumors to study therapeutic response. STAR Protoc..

[86] Sullivan KM, Jiang X, Guha P, Lausted C, Carter JA, Hsu C (2023). Blockade of interleukin 10 potentiates antitumour immune function in human colorectal cancer liver metastases. Gut.

[87] Relph K, Annels N, Smith C, Kostalas M, Pandha H (2020). Oncolytic Immunotherapy for Bladder Cancer Using Coxsackie A21 Virus: Using a Bladder Tumor Precision-Cut Slice Model System to Assess Viral Efficacy. Methods Mol Biol.

[88] Kusmartsev S, Kwenda E, Dominguez-Gutierrez PR, Crispen PL, O'Malley P (2022). High Levels of PD-L1+ and Hyal2+ Myeloid-derived Suppressor Cells in Renal Cell Carcinoma. J Kidney Cancer VHL.

[89] Salas A, López J, Reyes R, Évora C, de Oca FM, Báez D (2020). Organotypic culture as a research and preclinical model to study uterine leiomyomas. Sci Rep.

[90] Naipal KAT, Verkaik NS, Sánchez H, van Deurzen CHM, den Bakker MA, Hoeijmakers JHJ (2016). Tumor slice culture system to assess drug response of primary breast cancer. BMC Cancer.

[91] Holliday DL, Moss MA, Pollock S, Lane S, Shaaban AM, Millican-Slater R (2013). The practicalities of using tissue slices as preclinical organotypic breast cancer models. J Clin Pathol.

[92] Kleinhans R, Brischwein M, Wang P, Becker B, Demmel F, Schwarzenberger T (2012). Sensor-based cell and tissue screening for personalized cancer chemotherapy. Med Biol Eng Comput.

[93] Vesci L, Carollo V, Roscilli G, Aurisicchio L, Ferrara FF, Spagnoli L, de Santis R (2015). Trastuzumab and docetaxel in a preclinical organotypic breast cancer model using tissue slices from mammary fat pad: Translational relevance. Oncol Rep.

[94] Chakrabarty S, Quiros-Solano WF, Kuijten MMP, Haspels B, Mallya S, Lo CSY (2022). A Microfluidic Cancer-on-Chip Platform Predicts Drug Response Using Organotypic Tumor Slice Culture. Cancer Res.

[95] Capala ME, Pachler KS, Lauwers I, de Korte MA, Verkaik NS, Mast H (2023). Ex Vivo Functional Assay for Evaluating Treatment Response in Tumor Tissue of Head and Neck Squamous Cell Carcinoma. Cancers (Basel).

[96] Humpel C (2015). Organotypic brain slice cultures: A review. Neuroscience.

[97] Jimenez-Valdes RJ, Can UI, Niemeyer BF, Benam KH (2020). Where We Stand: Lung Organotypic Living Systems That Emulate Human-Relevant Host-Environment/Pathogen Interactions. Front Bioeng Biotechnol.

[98] Dewyse L, Reynaert H, van Grunsven LA (2021). Best Practices and Progress in Precision-Cut Liver Slice Cultures. Int J Mol Sci.

[99] Majorova D, Atkins E, Martineau H, Vokral I, Oosterhuis D, Olinga P (2021). Use of Precision-Cut Tissue Slices as a Translational Model to Study Host-Pathogen Interaction. Front Vet Sci..

[100] Smith RE (1970). Comparative evaluation of 2 instruments and procedures to cut nonfrozen sections. J Histochem Cytochem.

[101] Atkins AG, Xu X, Jeronimidis G (2004). Cutting, by ‘pressing and slicing’, of thin floppy slices of materials illustrated by experiments on cheddar cheese and salami. J Mater Sci.

[102] Reyssat E, Tallinen T, Le Merrer M, Mahadevan L (2012). Slicing softly with shear. Phys Rev Lett..

[103] Christensen JR, Larsen KB, Lisanby SH, Scalia J, Arango V, Dwork AJ, Pakkenberg B (2007). Neocortical and hippocampal neuron and glial cell numbers in the rhesus monkey. Anat Rec (Hoboken).

[104] Mattei G, Cristiani I, Magliaro C, Ahluwalia A (2015). Profile analysis of hepatic porcine and murine brain tissue slices obtained with a vibratome. PeerJ..

[105] Farniev VM, Shmelev ME, Shved NA, Gulaia VS, Biktimirov AR, Zhizhchenko AY (2022). Nanomechanical and Morphological AFM Mapping of Normal Tissues and Tumors on Live Brain Slices Using Specially Designed Embedding Matrix and Laser-Shaped Cantilevers. Biomedicines.

[106] Ragan T, Kadiri LR, Venkataraju KU, Bahlmann K, Sutin J, Taranda J (2012). Serial two-photon tomography for automated ex vivo mouse brain imaging. Nat Methods.

[107] Economo MN, Clack NG, Lavis LD, Gerfen CR, Svoboda K, Myers EW, Chandrashekar J (2016). A platform for brain-wide imaging and reconstruction of individual neurons. Elife..

[108] Wang J, Li C, Chen S-C (2019). Sectioning soft materials with an oscillating blade. Precis Eng.

[109] Li Y, Ding Z, Deng L, Fan G, Zhang Q, Gong H, et al. Precision vibratome for high-speed ultrathin biotissue cutting and organ-wide imaging. iScience. 2021;24:103016. doi:10.1016/j.isci.2021.103016. 10.1016/j.isci.2021.103016 PMC8426277 34522859

[110] Pesce L, Scardigli M, Gavryusev V, Laurino A, Mazzamuto G, Brady N (2022). 3D molecular phenotyping of cleared human brain tissues with light-sheet fluorescence microscopy. Commun Biol.

[111] Mendez OA, Flores Machado E, Lu J, Koshy AA (2021). Injection with Toxoplasma gondii protein affects neuron health and survival. Elife.

[112] Maurer M, Gresnigt MS, Last A, Wollny T, Berlinghof F, Pospich R (2019). A three-dimensional immunocompetent intestine-on-chip model as in vitro platform for functional and microbial interaction studies. Biomaterials..

[113] Thomas DP, Zhang J, Nguyen N-T, Ta HT (2023). Microfluidic Gut-on-a-Chip: Fundamentals and Challenges Biosensors (Basel).

[114] Kim H, Hong S-H, Jeong HE, Han S, Ahn J, Kim J-A (2022). Microfluidic model for in vitro acute Toxoplasma gondii infection and transendothelial migration. Sci Rep.

[115] Burgstaller G, Sengupta A, Vierkotten S, Preissler G, Lindner M, Behr J (2018). Distinct niches within the extracellular matrix dictate fibroblast function in (cell free) 3D lung tissue cultures. Am J Physiol Lung Cell Mol Physiol.

[116] Kim SY, Mongey R, Griffiths M, Hind M, Dean CH (2020). An Ex Vivo Acid Injury and Repair (AIR) Model Using Precision-Cut Lung Slices to Understand Lung Injury and Repair. Curr Protoc Mouse Biol..

[117] Bartucci R, van der Meer AZ, Boersma YL, Olinga P, Salvati A (2021). Nanoparticle-induced inflammation and fibrosis in ex vivo murine precision-cut liver slices and effects of nanoparticle exposure conditions. Arch Toxicol.

[118] Fahy GM, Guan N, de Graaf IAM, Tan Y, Griffin L, Groothuis GMM (2013). Cryopreservation of precision-cut tissue slices. Xenobiotica.

[119] Zhang Y, Wang Z-Y, Jing H-S, Zhang H-D, Yan H-X, Fan J-X, Zhai B (2022). A pre-clinical model combining cryopreservation technique with precision-cut slice culture method to assess the in vitro drug response of hepatocellular carcinoma. Int J Mol Med.

[120] Patel VS, Amin K, Wahab A, Marimoutou M, Ukishima L, Alvarez J (2023). Cryopreserved human precision-cut lung slices provide an immune competent pulmonary test system for "on-demand" use and long-term cultures. Toxicol Sci.

